# Using Morphogenic Genes to Improve Recovery and Regeneration of Transgenic Plants

**DOI:** 10.3390/plants8020038

**Published:** 2019-02-11

**Authors:** Bill Gordon-Kamm, Nagesh Sardesai, Maren Arling, Keith Lowe, George Hoerster, Scott Betts, Todd Jones

**Affiliations:** Corteva Agriscience™, Agriculture Division of DowDuPont, Johnston, IA 50131, USA; nagesh.sardesai@corteva.com (N.S.); maren.arling@pioneer.com (M.A.); keith.lowe@pioneer.com (K.L.); george.hoerster@pioneer.com (G.H.); scott.betts@pioneer.com (S.B.); todd.j.jones@pioneer.com (T.J.)

**Keywords:** transformation, morphogenic, embryogenesis, meristem formation, organogenesis

## Abstract

Efficient transformation of numerous important crops remains a challenge, due predominantly to our inability to stimulate growth of transgenic cells capable of producing plants. For years, this difficulty has been partially addressed by tissue culture strategies that improve regeneration either through somatic embryogenesis or meristem formation. Identification of genes involved in these developmental processes, designated here as morphogenic genes, provides useful tools in transformation research. In species from eudicots and cereals to gymnosperms, ectopic overexpression of genes involved in either embryo or meristem development has been used to stimulate growth of transgenic plants. However, many of these genes produce pleiotropic deleterious phenotypes. To mitigate this, research has been focusing on ways to take advantage of growth-stimulating morphogenic genes while later restricting or eliminating their expression in the plant. Methods of controlling ectopic overexpression include the use of transient expression, inducible promoters, tissue-specific promoters, and excision of the morphogenic genes. These methods of controlling morphogenic gene expression have been demonstrated in a variety of important crops. Here, we provide a review that highlights how ectopic overexpression of genes involved in morphogenesis has been used to improve transformation efficiencies, which is facilitating transformation of numerous recalcitrant crops. The use of morphogenic genes may help to alleviate one of the bottlenecks currently slowing progress in plant genome modification.

## 1. Introduction

Despite progress in crop transformation over the past several decades, efficient production of transgenic plants remains one of the major barriers to crop improvement [1]. There are two components to producing transgenic plants. The first is the ability to introduce and express transgenes (transformation), and the second is the ability to form tissue (typically de novo embryos or shoots) capable of regenerating into a fertile plant. Many plant species (or genotypes) remain difficult to transform and regenerate. Such varieties are referred to as being recalcitrant to transformation and plant regeneration. One of the promising tools helping to reduce this recalcitrance (and thus alleviate the bottleneck) is the use of genes involved in controlling plant growth and development.

Morphogenesis, or the organized spatial development of embryos, tissues, and organs, is a tightly controlled process involving networks of genes acting sequentially or in concert. Within this broad context, the concept of trying to use genes involved in either embryogenesis or meristem maintenance has attracted the attention of plant transformation researchers for many years. Basic research within these two well-defined areas has contributed an ever-expanding number of genes and gene networks involved in embryo development [2] and meristem development [3,4,5,6] that we will not attempt to cover here. De novo regeneration of plants typically occurs through either somatic embryogenesis or organogenesis (de novo formation of new meristems or through rearrangement of pre-existing meristems), traditionally manipulated by adjusting auxin/cytokinin ratios in the medium [6,7,8]. In addition to exogenous hormone manipulation, ectopic overexpression of plant genes that control growth and development has also proven to be useful.

Of course, there are numerous reports where non-plant genes have been used to improve transformation frequencies and/or plant regeneration. Examples include tumor-inducing genes from *Agrobacterium*, such as the isopentyl transferase or *ipt* gene [9,10], *rolC* [11,12], *rolB*, *6B* [13], and *tzs* [14,15], and viral genes that stimulate the plant cell cycle [16]. Further details for non-plant genes are beyond the scope of this review. Instead, we will focus on research that has demonstrated a potentially useful morphogenic growth response due to ectopic overexpression of a plant embryo or meristem gene (morphogenic genes), or has demonstrated a practical benefit for plant transformation or regeneration. Plant transformation and regeneration improvements can be further distinguished by their impact on either improving transformation efficiencies, or improving the regeneration process to recover transgenic plants. Finally, we are making a distinction between reports focused solely on observations of morphogenic responses, and those studies that describe practical methods to improve transformation and/or regeneration. Both types of results are important in terms of their contribution to the field of transformation research, and are described below.

**Figure 1:**
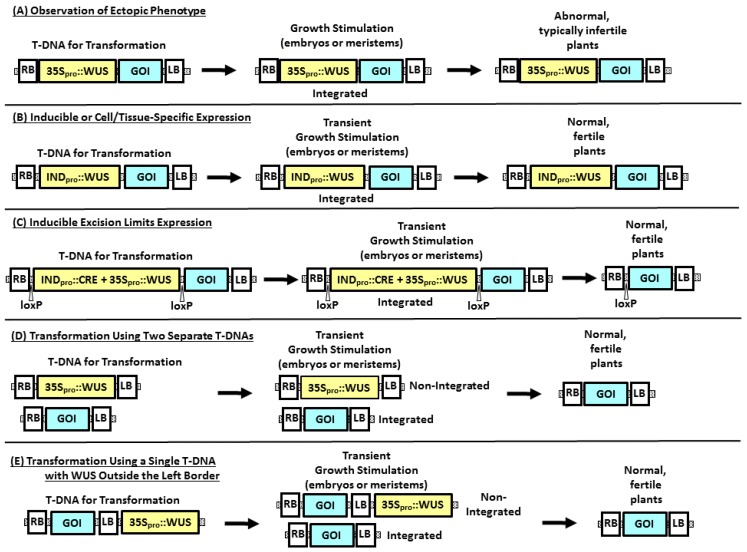
Methods for expression of morphogenic genes in plant transformation. WUS is used to exemplify the morphogenic gene expression cassettes, which could be designed for overexpression of the gene, downregulation of a gene, or combinations of genes, while the box labeled “GOI” (Genes Of Interest) represents trait gene expression cassettes. (**A**) Using a constitutive promoter, such as CaMV35S, to drive expression of a WUS gene results in growth stimulation of cells transformed with the T-DNA, either through somatic embryogenesis or through meristem proliferation. (**B**) Using an inducible promoter to drive expression of WUS will result in growth stimulation only when the plant tissue is exposed to the inducing stimulus (typically a chemical ligand). (**C**) Growth stimulation can also be effectively controlled by using a combination of constitutive expression of WUS and inducible expression of CRE recombinase to remove the WUS expression cassette. (**D**) Transforming the same plant cell with a T-DNA containing the WUS expression cassette and a second T-DNA containing the trait expression cassette will also provide transient growth stimulation sufficient to recover regenerable tissues, such as somatic embryos, without the integration of the morphogenic gene. (**E**) Using a single T-DNA containing the trait, with the WUS expression cassette outside the T-DNA Left Border sequence, higher numbers of the trait-containing T-DNA are introduced relative to the low numbers of “read-through” sequences containing the WUS gene, providing transient growth stimulation without WUS integration. IND_pro_ is used to represent chemically inducible promoters, such as the estradiol-, glucocorticoid-, or tetracycline-responsive promoters in 1-B. Promoters that are induced by physical conditions, such as desiccation (e.g., the RAB17 promoter in [17]), are used to control recombinase-mediated excision (as in 1-C). CRE represents the CRE recombinase expression cassette and loxP are the CRE-recombinase target sites. RB and LB represent the right and left T-DNA border sequences, respectively.

Characterizing morphogenic genes involves phenotypic analysis of knockout mutants and/or transgenic experiments typically involving either ectopic overexpression or downregulation in plants. Often, the two strategies are combined, where introduction of an expression cassette is used to complement a mutant phenotype. Morphogenic responses have been observed using transient expression, constitutive expression, or induction of gene expression by exposure of a stable transgenic event to a chemical ligand. Using a plant morphogenic gene to improve transformation, on the other hand, almost invariably involves limiting the length of time that expression occurs to avoid later pleiotropic effects in regenerated T0 plants and subsequent progeny generations (Figure 1).

## 2. Phenotypic Responses from Ectopic Overexpression of Morphogenic Genes

The genes involved in embryogenesis, meristem maintenance, and hormone metabolism are numerous and have been studied for many years [2,3,4,5,6]. Within this large and ever-expanding body of literature, there has been a steady stream of reports demonstrating morphogenesis in response to altered expression of these genes. These observations provide the groundwork that inspires new strategies for transformation research, and are discussed below.

Numerous genes mentioned in this review are typically categorized as genes involved in embryogenesis, meristem function, or hormone pathways. However, we have chosen to group these genes based on their practical benefit (or potential) when used for transformation. Therefore, morphogenic genes that have stimulated an embryogenic or meristematic response when overexpressed were grouped into two categories based on the observed growth response: A) those that enhance a pre-existing embryogenic response under conditions (media composition, exogenous hormones, or even the tissue type) that already elicit the growth response, and B) those that produce ectopic somatic embryos or meristems under conditions where such a response is typically not observed (see Table 1 for a list of genes).

### 2.1. Enhancing the Somatic Embryogenic Response

In this category, overexpression of the plant gene results in enhanced formation of somatic embryos under in vitro culture conditions in which somatic embryogenesis already occurs (see Table 1, Strategy A). This includes the observation that when *SOMATIC EMBRYOGENESIS RECEPTOR KINASE1* (*SERK1*), a gene normally associated with anther and pollen development, was overexpressed using the Cauliflower Mosaic Virus 35S promoter (CaMV 35S) in *Arabidopsis thaliana*, no changes in plant phenotype were observed, but the embryogenic callus response was improved 3-4-fold over wild-type [18]. This demonstrated that SERK1 stimulated an enhanced somatic embryo response from germinating seedlings placed on media capable of eliciting this response already. Similarly, overexpression of the *Coffea canephora SERK1* gene during the in vitro somatic embryogenesis process enhanced the production of somatic embryos by 2-fold, while silencing the gene dramatically reduced the somatic embryogenesis response [19]. Similar conclusions have been reached in reports in which the *AGAMOUS-LIKE15* (*AGL15*) gene was overexpressed, enhancing the formation of secondary somatic embryos from cultured zygotic embryos in Arabidopsis [20], increasing the number of somatic embryos in soybean cultures [21], and again enhancing production of embryogenic callus in cotton [22]. The similarities between SERK1 and AGL15 overexpression are not surprising, since AGL15 is part of the SERK1 protein complex [58]. Interestingly, SERK1 and SERK3 have been shown to be co-receptors, along with BRASSINOSTEROID INSENSITIVE 1 protein (BRI1), of the brassinosteroid class of plant growth regulators [59,60], and the SERK proteins and BRI1 phosphorylate one another upon brassinosteroid sensing. Based on protein structure, the SERK proteins appear to mediate brassinosteroid signaling across the plasma membrane [61]. This observation makes an intriguing connection between brassinosteroid response and embryogenesis.

Increased embryogenic responses have also been reported using genes more typically associated with meristem formation, as with *A. thaliana WUSCHEL* (*AtWUS*), a key regulator of meristem cell fate [62], or *SHOOT MERISTEMLESS* (*STM*), which is required for proper meristem formation [63]. In transgenic *Coffea canephora* containing an estradiol-inducible *AtWUS* construct, leaf discs placed on estradiol increased somatic embryo formation from a control level of one somatic embryo per leaf segment (non-treated), up to a level of 3–5 somatic embryos per transgenic leaf segment after estradiol exposure [23]. Shortly after, it was reported that constitutive overexpression (using the CaMV 35S promoter) of the *Brassica napus*, *Brassica oleracea*, or *Brassica rapa homologs of STM* in *Arabidopsis thaliana* cotyledons placed on auxin-containing medium resulted in an approximately two-fold increase of somatic embryo formation relative to the wild-type control. In transgenic *B. napus* containing the 35S::*BnSTM* construct, a similar two-fold increase was observed in microspore-derived embryogenesis [24]. Similarly, in experiments with the objective of improving transformation methods in cotton (*Gossypium hirsutum* L.), the 35S::*AtWUS* cassette was introduced into hypocotyl segments and a three-fold increase in the formation of somatic embryos was observed [25]. Further, the somatic embryos derived from the WUS treatment (when the *WUS* gene is being overexpressed) produced leaf-like structures but failed to regenerate into plants, likely due to the deleterious effect of WUS ectopic overexpression on subsequent regeneration.

### 2.2. Ectopic Formation of Somatic Embryos or Meristems

In the second category, overexpression of the plant gene results in direct ectopic formation or spontaneous formation/acquisition of structures resembling embryos (often with embryo characteristics, such as increased oil levels) or meristems in the absence of inductive conditions (see Table 1, Strategy B). In 2002, two important milestone research articles were published that characterized embryonic morphogenesis as a result of ectopic overexpression of either the *Brassica napus BABY BOOM* (*BnBBM*) gene [26] or the *AtWUS* gene [38]. We will first review *BBM* and other genes involved in embryogenesis, and then later turn our attention to genes involved in meristem function. The *BBM* gene, a member of the AP2/ERF superfamily of transcription factors [64], has generated great interest among transformation researchers from the first publication [26]. In these experiments, it was observed that constitutive expression of the Brassica *BBM* gene in Arabidopsis resulted in ectopic somatic embryo formation in vegetative portions of progeny plants, for example in the shoot apex and leaves, and these ectopic somatic embryos could in turn produce plants in the absence of hormones. These results stimulated further research aimed at harnessing BBM-induced somatic embryogenesis to aid in the recovery of transgenic T0 plants.

This first publication was followed by reports in other plant species or using orthologs of the *BnBBM* gene, providing additional insights into how *BBM* worked. Srinivasan et al. [27] investigated the ability of various BBM orthologs to induce embryogenic responses in a less-related species. Spontaneous somatic embryogenesis was not observed using 35S::*AtBBM* in *Nicotiana tabacum* [27]. However, when these authors used a steroid-inducible, post-translationally controlled AtBBM fusion protein (*AtBBM*~GR) regulated by the 35S promoter to create stable lines and evaluated progeny, spontaneous ectopic shoot and root formation was observed upon addition of the inducing ligand dexamethasone (DEX). Further, when hypocotyls were exposed to DEX, somatic embryos could be induced when the growth medium contained either zeatin or benzylaminopurine. While the authors attributed the difference observed between Arabidopsis and tobacco to varying competence in response to the BBM signal, they also pointed out that expression of *BBM* from either Arabidopsis or Brassica in tobacco could produce developmental responses that differ from those observed using the endogenous tobacco *BBM* gene.

In another example of expressing an orthologous *BBM* gene, a constitutively expressed soybean gene (35S::*GmBBM*) was transformed into Arabidopsis [28], and ectopic somatic embryos were observed growing from the cotyledons, the shoot apical meristem, and the hypocotyls of stably transformed plants. Again, differences were observed in the pattern of somatic embryo formation, but, in general, the three studies provide strong evidence that constitutive expression of BBM can result in ectopic somatic embryo formation. Using the genomic clone of *Theobroma cacao BBM* (*TcBBM*) under the control of the 35S promoter, Florez et al. [29] demonstrated that it phenocopied the effects of *AtBBM* in Arabidopsis and stimulated the formation of somatic embryos from Theobroma cotyledons cultured on hormone-free media. Although somatic embryos were formed in cacao using *TcBBM*, constitutive expression prevented normal plant regeneration.

For many species, recovery of transgenic events is not the bottleneck, but instead regeneration of viable T0 plants is inefficient and rate-limiting. For example, when a *BBM* ortholog from oil palm (*Elaeis guineensis*) was cloned into an expression cassette behind the CaMV 35S promoter and then transformed into Arabidopsis, it was observed that cotyledon, leaf, or root segments from stable transgenic events exhibited enhanced rates of shoot formation relative to the wild-type controls [30]. These results are consistent with earlier observations where regeneration was improved through ectopic overexpression of BBM [26]. However, in all these reports, the 35S promoter was used to drive constitutive expression of the transgene and, as a result, no data on recovery of mature fertile plants were presented.

Tsuwamoto et al. [31] using Arabidopsis *EMBRYOMAKER* (*AtEMK*), a gene related to *BBM* (both within the AP2/ERF superfamily) driven by the CaMV 35S promoter in Arabidopsis, produced transgenic progeny that could be phenotypically evaluated. In these experiments, ectopic overexpression of *AtEMK* produced light-green embryo-like structures (possessing morphological and/or biochemical characteristics normally observed in zygotic embryos but lacking the full functionality of being able to develop into a plant) at the tip of cotyledons in 23% of the seedlings. While several of the embryo-like structures developed small features resembling roots and leaves, these outgrowths did not continue to develop. As ectopic overexpression of *AtEMK* resulted in pleiotropic effects, it may be necessary to express *AtEMK* under a regulated system for obtaining normal plantlets from somatic embryos and the embryo-like structures.

Another gene observed to function during early embryo development in Arabidopsis is *RKD4*, a member of the RWP-RK transcription factor family essential for the first asymmetrical division of the zygote to form the two cells that will give rise to the embryo and suspensor [65,66]. Following up on these characterizations of RKD4 function, Mursyanti et al. [32] demonstrated in orchid (the hybrid *Phalaenopsis* “Solo Vivien”) that chemical induction of transgenic *RKD4* in leaf tissue resulted in ectopic somatic embryogenesis, a very exciting observation in a species normally reluctant to produce direct somatic embryos (de novo embryos that arise from somatic cells, having the capacity to develop into plants).

Genes normally involved in embryo maturation, such as *LEAFY COTYLEDON1* (*LEC1*), *LEAFY COTYLEDON2* (*LEC2*), and *FUSCA3* (*FUS3*), produce similar morphogenic responses when overexpressed. The first of these genes characterized in Arabidopsis was *LEC1* by Lotan et al. [33], in which a 35S::*AtLEC1* cassette was introduced into Arabidopsis using *Agrobacterium*. Progeny seed were germinated and embryo-like structures were observed in many germinating plantlets. For example, cotyledon-like structures were observed to replace what should have been the first true leaves of the seedling. The embryo-like nature of these tissues was corroborated by other embryo characteristics, such as accumulation of cruciferin-A storage protein and oleosin RNAs. However, despite forming embryo-like structures, no functional ectopic somatic embryos were observed (i.e., embryo formation was incomplete).

In a similar study, a *Citrus sinensis LEC1* paralog called *L1L* (*LEC1-Like*) was constitutively overexpressed in a 35S::*CsL1L* cassette after transformation of “Olinda” sweet orange or “Guoqing No. 1” Satsuma mandarin epicotyls using *Agrobacterium* [34]. In these experiments, the authors observed that the normally recalcitrant epicotyls formed some embryo-like structures after one month, and after another two months on elongation medium formed shoots with aberrant leaves. This suggests that in Citrus, *L1L* overexpression is sufficient to produce functional somatic embryos.

Uddenberg et al. [35] observed that overexpression of the *PaHAP3A* (a *LEC1*/*L1L* gene from Norway spruce, based on sequence information) did not result in ectopic somatic embryo formation in vegetative tissues. However, when expression was induced during zygotic embryo maturation, ectopic somatic embryos formed on the surface of the zygotic embryos. As noted by Srinivasan et al. [27] for BBM, this suggests that certain cell types may be more receptive to inductive signals, such as that being provided by the PaHAP3A protein [35].

In a variation on the general theme of using the CaMV 35S promoter to drive constitutive expression, Gazzarrini et al. [36] used an epidermal-specific promoter Meristem Layer1 (ML1) from Arabidopsis to drive expression of the Arabidopsis *FUSCA* gene (*AtFUS3*). Consistent with previous *LEC1* results, ectopic overexpression of *AtFUS3* resulted in the formation of cotyledon-like leaves that accumulated storage protein bodies (similar to the cells in an embryo). This observation is similar to phenotypes observed with *LEC1* overexpression.

In experiments similar to those described above, stable transgenic lines were produced by introducing 35S::*AtLEC2* into *lec2-1* and *lec2-5* mutant lines of Arabidopsis, and ectopic somatic embryos formed that were competent to germinate and produce plants [37]. However, the resultant plant phenotypes were aberrant and the authors did not comment on fertility. Nonetheless, it appears that *LEC2* might result in more complete somatic embryo formation, compared to either *LEC1*, *L1L*, or *FUS3*. This is consistent with evidence indicating that LEC2 functions upstream of and activates both *LEC1* and *FUS3* [67], in which case the penetrance of the somatic embryo phenotype might be stronger in plants overexpressing *LEC2*.

Similar to observations in which overexpression of genes involved in meristem initiation and maintenance have increased a pre-existing embryogenic response [19,20,21,22,24,25,26], their ectopic overexpression has also been reported to stimulate the formation of somatic embryos where they would otherwise not be observed.

The first report of a “meristem” gene stimulating embryo formation was by Zuo et al. [38], who obtained an estradiol-induced activation-tagged *pga6-1* mutant line in Arabidopsis that formed somatic embryos from root tips. This was confirmed to be *AtWUS*, which phenocopied the original activation-tagged mutant when expressed using either the estradiol-inducible or 35S promoters. Somatic embryo formation was also observed from a variety of tissues with de novo embryos from root tips being the most common observation. As with the observations by Boutilier et al. [26] with BBM, these results clearly demonstrate that WUS can stimulate the vegetative-to-embryonic transition.

By 2002 it was well-established that the proper expression of numerous genes was essential for morphogenesis. In keeping with this concept, Gallois et al. [39] analyzed the impact that two such genes might have on ectopic meristem initiation, using two different methods to control expression of *STM* and *WUS* in Arabidopsis (chemical induction and heat shock, respectively). Based on their observations of treated leaf tissue, the authors hypothesized that *STM* and *WUS* expression would produce clusters of cells adjacent to the WUS foci that represented incipient meristems (confirmed by a meristem-specific biomarker). These young ectopic meristems were initiated, but self-perpetuating meristems were not established.

In another interesting paper by Gallois et al. [40], stable transgenic lines were produced in Arabidopsis where *WUS* expression was activated through either HSP::*CRE*-mediated excision, or a GAL4-VP16 activation system. Upon either type of WUS activation, unique phenotypes were observed in root tips, and the type of response was dependent on other variables, such as hormone regime or co-expression of another morphogenic transcription factor. For example, when *WUS* was expressed alone in hormone-free medium, ectopic shoots and leaves were observed. When WUS was expressed in the presence of exogenous auxin (2,4-D), ectopic somatic embryos were formed. Finally, floral structures were observed when *WUS* was induced along with a constitutively expressed *LEAFY* gene, a master regulator of floral development [68].

Despite somatic embryogenesis from root tips being consistently observed when *WUS* expression was induced in Arabidopsis, estradiol induction of *AtWUS* in *Nicotiana tabacum* resulted in a direct organogenic response, where the root tips became swollen and developed green shoots rather than somatic embryos [41]. When *AtWOX5* (a member of the *WUS*/*WOX* gene family expressed in the root tip) was substituted in these experiments, root tip swelling and green shoot formation were again observed [42].

While Gallois et al. [39] focused on the interaction of WUS and STM in Arabidopsis, the impact of the maize STM ortholog *KNOTTED1* (*KN1*) has also been reported. When the maize *KN1* gene was constitutively overexpressed (35S::*ZmKN1*) in *Nicotiana tabacum*, it resulted in a 3-fold increase in shoot organogenesis, relative to the NPTII-only control [43]. The increase in shoots in the *KN1* treatment was obtained with no antibiotic or herbicide selection and no exogenous hormones in the media, and the resultant plants were bushy, with altered leaf morphology and underdeveloped roots.

The overexpression of *KN1* (using the CaMV 35S promoter) can also bypass an intermediate callus phase, as reported by Nishimura et al. [44]. Constitutive expression of tobacco KN1 orthologs in *Nicotiana tabacum* resulted in a range of pleiotropic phenotypes. Transgenic plants containing the *NTH20* (a knotted-like homeobox gene) expression cassette occasionally produced ectopic shoot meristems that would develop into small shoots with leaves emerging from the original leaf surface.

Other genes that play a role in shoot meristem formation are the *CUP-SHAPED COTYLEDON* genes *CUC1* and *CUC2*. Using the Arabidopsis *CUC1* and *CUC2* genes, Daimon et al. [45] showed that overexpression of these genes under a strong promoter (CaMV 35S) led to the rapid production of adventitious shoots in transgenic calli derived from Arabidopsis hypocotyls. *CUC1*- and *CUC2*- overexpressing calli produced an average of 4.8 and 3.3 adventitious shoots per callus, respectively, while the controls produced 0.5 shoots that developed more slowly. In the absence of the phytohormones, no adventitious shoots were formed, indicating that *CUC1* and *CUC2* function was hormone-dependent.

In the context of using plant genes to improve plant transformation, genes involved in hormone signal transduction (whether receptors or downstream targets) also fall under our ’morphogenic’ classification. Two such candidates are the Arabidopsis *ENHANCER OF SHOOT REGENERATION* genes (*ESR1* and *ESR2*), both identified through mutant screening and demonstrated to be involved in the cytokinin response pathway. As part of this characterization, it was demonstrated that overexpression of both *ESR1* and *ESR2* conferred cytokinin-independent shoot formation [47,48]. The Arabidopsis auxin-response gene *MONOPTEROS* (*MP*) is also of interest in this respect. In experiments focusing on the role of the *MP* gene on shoot formation, it was observed that using the endogenous promoter to drive expression of a C-terminally deleted gene referred to as *MPΔ* (lacking the domain involved in auxin/IAA interactions) increased the formation of shoot apical meristems from callus [49].

In addition to genes involved in the hormone signaling pathway, levels of hormones can affect the phenotypic response of genes involved in morphogenesis. Transgenic Arabidopsis explants overexpressing *LEC2* under a DEX-inducible system produced somatic embryos in the presence of low auxin concentrations, while increasing the concentration resulted in the production of calli [46].

## 3. Strategies to Improve Transformation Using Morphogenic Genes

Given the focus of this review, it is easy to see why researchers in plant transformation would view the genes described above as potential tools in the transformation process. However, constitutive and strong expression of these morphogenic genes often caused undesired pleiotropic effects, including reduced fertility. In the examples discussed in this section, constitutive expression of the morphogenic genes (Figure 1A) or inducible expression in stably transformed plants (Figure 1B) were the predominant strategies. To render true utility, an additional step is required: combining optimized expression of the plant morphogenic genes being used with a robust method to limit expression after plant transformation/regeneration has occurred and their utility has expired. Such methods typically demonstrate improved transformation efficiency, enhanced regeneration, or both in a manner that produces healthy, fertile T0 plants. It should also be emphasized that to characterize gain-of-function phenotypes, as in many of the examples above, stable transgenic germplasm was produced using conventional selection methods (for example, after floral-dip transformation in Arabidopsis). As a result, the ectopic phenotype was evaluated in homogeneously transgenic tissues in seed-derived plants. This contrasts with expressing a morphogenic gene in a single cell surrounded by wild-type tissue, as when trying to use a morphogenic gene to recover transgenic events. In this situation, differential expression of the morphogenic gene provides a positive growth advantage or an identifiable phenotype (relative to wild-type cells) that can be used for selection. This is the case in the examples described below (see Table 1, Strategy C).

There are several reports that describe ectopic overexpression of morphogenic genes; however, deleterious pleiotropic phenotypes were observed in plants when constitutively expressed. Studies on how to control the timing and level of expression for these genes through downregulation or elimination have lagged. To date, we can identify four approaches to address this problem in the literature: (i) stimulating the morphogenic growth response through inducible expression of the morphogenic gene (Figure 1B) followed by removal of the inducing ligand to turn off expression, (ii) excision of the plant morphogenic gene (Figure 1C) when no longer needed, (iii) use of a plant promoter that turns off when no longer needed to permit normal growth and reproduction in transgenic plants, and (iv) using Agrobacterium-mediated delivery in a manner that favors transient expression of the morphogenic genes (Figure 1D,E).

Inducible expression has provided a robust method for using morphogenic genes to recover fertile transgenic plants. There are few reports on the transformation of recalcitrant species, and thus the first case presented here of using an inducible morphogenic gene for improving transformation and regeneration deserves emphasis. Pepper varieties (*Capsicum annuum*) have very poor transformation efficiencies and regenerative capacity. Heidmann et al. [50] transformed cotyledon explants of two sweet pepper varieties with a 35S::*BnBBM*~GR construct and cultured the explants on media supplemented with thidiazuron and DEX for 2 months. Emerging shoots were transferred to DEX-free elongation medium for 4 weeks and then pre-rooting medium for a month. Transformation efficiency with the regulated *BBM* expression was >1% compared to 0% with a 35S::*GUS* (*β-glucuronidase*) construct. This is an important step forward in what has historically been a very recalcitrant crop.

Also using the DEX-inducible *AtBBM*~GR system, Lutz and colleagues [51] described a method to obtain fertile, transgenic Arabidopsis plants from leaf cultures. In the absence of auxins, transgenic leaf explants produced prolific shoots in around 4 weeks in the presence of DEX, while explants on medium without the ligand did not produce shoots, and explants from wild-type plants became necrotic, irrespective of whether DEX was added or not. Recovered shoots were further cut into smaller segments and regenerated on medium containing DEX for 3 months. Removing the shoots from the DEX-containing medium allowed for regeneration of plantlets with normal flowering and seed formation over 3 months’ time. Fertile transgenic progeny were produced from the collected seeds.

Using the estradiol-inducible system [38], Wang et al. [52] identified *PLANT GROWTH ACTIVATION* genes, such as *PGA37*, which resulted in a vegetative-to-embryogenic transition when overexpressed in Arabidopsis. While the downstream targets have yet to be characterized, *PGA37* was determined to encode a MYB118 transcription factor based on structural similarities within the DNA-binding domain. When *PGA37* was expressed under inducible control, somatic embryos developed from root explants and was associated with increased *LEC1* expression. Estradiol-induced expression of *PGA37* in the presence of auxin produced green-yellowish embryonic calli in 7–10 days and generated somatic embryos upon culturing for 3–5 weeks. Upon removal of estradiol from the medium (thus downregulating *PGA37* expression), the somatic embryos developed into healthy, fertile plantlets. Overexpression of a closely related homolog, *MYB115*, under the estradiol inducible system also led to the formation of somatic embryos from root explants.

When transforming recalcitrant species, such as trees, that in addition have maturation periods running into years, direct production of somatic embryos offers a relatively fast way for genetic manipulation [69]. One such example is use of the morphogenic gene *LEC2* to generate transgenic plants in *Theobroma cacao*, as described by Shires and colleagues [53]. They identified the *TcLEC2* transcription factor sequence and cloned it as a translational fusion with GR (the Glucocorticoid Receptor) driven by a CaMV 35S promoter. This DEX-inducible construct was used to transform cotyledon tissue of the variety Scavina-6 and cultured for about 6 months to screen for transgenic somatic embryos. Consistent with the transformation recalcitrance in *T. cacao*, only one transgenic embryo was recovered. This was proliferated by segmenting the cotyledons into several pieces to produce clonal-transformed somatic embryos. When tissue from these secondary embryos was placed on hormone-free media supplemented with DEX, multiple embryos were formed in 6 days. Levels of DEX up to 50 µM produced the most embryos (403) per 100 explants over a period of 4 months. In this experiment, a single somatic embryo was converted to a transgenic plant that developed normally. Young leaf tissue from the transgenic 35S::*TcLEC2*:GR plant was capable of prolific somatic embryo formation in the presence of DEX after 3 months, providing a promising method to regenerate secondary transgenics from leaf material.

In a recent publication using the estradiol-inducible system to control *WOX* gene expression, two combinations of Arabidopsis-derived *WOX* genes (*WOX2* + *WOX8* or *WOX2* + *WOX9*) were evaluated in the presence of 1 μM 2,4-D for 10 days. Both combinations resulted in substantial plantlet regeneration from *Nicotiana tabacum* leaf pieces, in contrast to the wild-type control where no plantlet regeneration was observed [54].

Excision-based strategies to control morphogenic gene expression are another alternative. The first demonstration of this concept in a dicot was reported in *Populus tomentosa* using *BBM* and FLP-recombinase for excision [55]. These authors designed a T-DNA construct consisting of a single pair of FRT (FLP Recombination Target Sites) flanking both a heat-shock inducible promoter driving FLP recombinase expression and a CaMV 35S promoter driving expression of the *Brassica campestris BBM* gene cassette. Using *Agrobacterium*-mediated transformation, 21 callus cultures of Chinese white poplar were transformed with this T-DNA and cultured on hormone-free medium. Six of the 21 calli developed a total of 12 somatic embryos approximately four weeks after transformation, and half of the somatic embryos germinated to form plantlets that had a dwarf phenotype with small wrinkled leaves when cultured for 60 days. Heat shock treatment at 42 °C for 2 hours led to excision of both the *FLP* recombinase and *BBM* cassettes in four of the six plants, resulting in reappearance of the normal phenotype in regenerated plants.

The first demonstration of using morphogenic genes followed by excision to improve monocot transformation has only recently been reported by Lowe et al. [17]. After *Agrobacterium*-mediated transformation of immature embryos of a normally recalcitrant Pioneer maize inbred, a strongly expressed *ZmBBM* (using the *Zea mays* UBIQUITIN promoter) plus a weakly expressed *ZmWUS2* (using the *Agrobacterium* NOPALINE SYNTHASE, or NOS, promoter) resulted in the stimulation of embryogenic callus. This combination of a weakly expressed *WUS2* gene and a strongly expressed *BBM* gene resulted in high transformation frequencies when immature embryos of the maize inbred were transformed. The callus was placed on dry filter paper for 3 days to stimulate a desiccation-induced maize promoter from an ABA-responsive gene (RAB17) driving *CRE* recombinase, which then efficiently excised all three expression cassettes. After excision of the *CRE*, *WUS2*, and *BBM* transgenes, all that remained in the integrated T-DNA locus were the genes of interest (for example, herbicide resistance and/or a visual marker gene). This also improved transformation efficiency in many other difficult or recalcitrant genotypes (difficult corn inbreds, sorghum, sugarcane, and Indica rice), and permitted transformation of previously non-transformable explants, such as mature embryo sections (starting with mature, dry seed) or leaf segments from 7–14-day-old seedlings. This method is beginning to facilitate enhanced transformation of previously recalcitrant public cereal varieties, such as the maize inbred B73 and the sorghum variety P898012 [56].

More recently, Lowe et al. [57] described an improved transformation system for maize using two new promoters: the maize AXIG1 promoter (auxin-inducible) driving *WUS2* and the maize PLTP promoter (PhosphoLipid Transfer Protein promoter) driving *BBM*. This new configuration of expression cassettes resulted in rapid formation of somatic embryos within 7 days, with germination of these newly formed somatic embryos producing plantlets ready for transplantation into soil and growth in the greenhouse within 21–30 days. Expression of both promoters was so low or confined to specific cell types (or tissues) in the plant that, even without excision of the PLTP::*BBM* and AXIG1::*WUS2* cassettes, the resultant T0 plants were all robust and fertile. This new rapid transformation system has worked in all Pioneer and public inbreds tested, as well as in recalcitrant sorghum and wheat varieties.

A recurring theme in all the transformation methods described above is the necessity of controlling expression of the morphogenic genes in order to recover normal-phenotype plants, through either inducible expression, developmentally regulated expression, or excision. In the case of inducible or developmentally regulated expression of *BBM* and *WUS2*, the morphogenic genes and trait genes are linked within the same construct. Alternatively, constructs designed for excision are larger and more complex, but the morphogenic genes are removed prior to plant regeneration.

However, two additional alternatives exist that avoid both pitfalls. In the first alternative, two T-DNAs are introduced from the same *Agrobacterium* (containing both plasmids). In the second alternative, two separate Agrobacteria are used to introduce different T-DNA plasmids. Using either strategy, the two transgenic loci are later segregated away from each other [70]. The basis for the second approach was illuminated in a report by Florez et al. [29], who demonstrated that transient expression of *TcBBM* delivered by *Agrobacterium* was sufficient to stimulate somatic embryo formation, with no indication of the transgenic *BBM* cassette being detected in the somatic embryos. The authors then speculated that a co-transfection technique could be used to obtain transgenic plants by mixing an *Agrobacterium* strain containing a *BBM* expression cassette along with a trait-containing strain.

One final variation on using *Agrobacterium* to deliver transient morphogenic gene activity relies on a commonly observed characteristic of T-DNA delivery, with many labs over the years reporting on the phenomenon of T-DNA border read-through [71,72,73,74]. As the name implies, when the T-DNA is processed in the *Agrobacterium* before delivery, inefficient T-strand processing will produce some percentage of T-DNA molecules that are not terminated precisely at the Left Border (LB) but continue to include sequence beyond the LB. As a means of reducing this type of unwanted read-through, researchers have employed negative selectable markers [72] positioned beyond the LB (outside the T-DNA) to eliminate plant cells that had integrated these sequences. Others have tried to exploit inefficient T-strand processing by positioning a positive marker gene beyond the LB that could be used transiently, producing selectable marker-free transgenic plants [75]. By placing an *Agrobacterium*-derived *IPT* gene outside the LB, many cells would receive a mixture of T-DNAs, with the majority of the T-strands processed properly (only containing the trait), but with a smaller percentage of T-strands not terminated properly at the LB thus containing the flanking *IPT* gene. In these cells, transient *IPT* expression would stimulate cytokinin production and shoot proliferation, and when the processed T-strand integrated and the *IPT*-containing T-strand did not, this would result in the recovery of trait-containing transgenic plants that contain no selectable marker.

Since it has not been reported that shoot proliferation in response to *IPT* expression occurs in maize and other cereal crops, we tested *WUS2* alone or *WUS2* + *BBM* for transient somatic embryo formation and subsequent germination to produce T0 plants by positioning these cassettes outside the LB [76]. This resulted in a simple non-excision method for using *WUS2* and *BBM* for transformation of recalcitrant maize genotypes that exploits inefficiencies of both T-strand processing and integration to allow for rapid transformation of maize while enriching for events with no *WUS2* or *BBM* integration.

For example, *Agrobacterium*-mediated T-DNA delivery into the Pioneer maize inbred PH1V69 is very efficient; however, without *BBM* and *WUS2* expression cassettes present in the T-DNA, it has so far proven impossible to recover transgenic T0 plants. Using inbred PH1V69, transformation was performed using an *Agrobacterium* strain LBA4404 that contained a single T-DNA plasmid [76]. Two versions were tested, with both containing two ‘Mock Trait’ expression cassettes within the T-DNA, a *Setaria italica*-derived UBIQUITIN promoter driving expression of a green fluorescent protein and an herbicide resistance cassette containing a *Sorghum bicolor* Acetolactate Synthase (ALS) promoter driving expression of *HRA* (a mutant maize *ALS* gene that confers resistance to sulfonylurea herbicides). For the first treatment, two expression cassettes were also positioned outside the LB of the T-DNA, with a PLTP promoter driving *WUS2*, and a PLTP promoter driving expression of *BBM* (*WUS2*/*BBM)*. In a second treatment, only the PLTP::*WUS2* was placed outside the LB (*WUS2* only). *Agrobacterium*-infection, resting, somatic embryo maturation, and regeneration were performed as described in Lowe et al. [57] and T0 plants were analyzed for the presence of the marker genes plus *WUS2* and *BBM* (when applicable) using qPCR. Starting with 196 immature embryos in each treatment, it was observed after analysis that the frequency of T0 plants (relative to the number of starting immature embryos) that were single-copy for marker genes and were negative for *WUS2* and *BBM* (when applicable) was 12.2% and 10.2% for the ‘*WUS2* only’ and ‘*WUS2*/*BBM*’ treatments, respectively. The percentage of T0 plants that contained either *BBM*/*WUS2* or *WUS2* alone (depending on the treatment) was 49% and 38%, respectively [76]. This suggests that while ‘read-through’ copies of *WUS2* or *WUS2*/*BBM* were clearly having a positive impact in terms of stimulating somatic embryo formation, there was also some unavoidable integration of T-DNA sequences that also carried along the flanking sequence (‘backbone’ from the *Agrobacterium* T-DNA-containing plasmid). This is expected and, consequently, the method requires PCR screening to identify perfect, single-copy T-DNA events. However, it should also be noted that the frequency of recovering perfect, single-copy events was comparable to that observed for the excision method for this inbred [17]. This represents a viable alternative to excision as a means of creating high-quality transgenic events in recalcitrant monocot crops that do not contain helper genes (in this case *WUS2* and *BBM*).

## 4. Conclusions

There has been meteoric progress in plant genome modification engendered by CRISPR/*CAS9* (CLUSTERED REGULARLY-INTERSPERSED SHORT PALINDROMIC REPEATS and the *CRISPR-ASSOCIATED 9* gene) over the past half-decade. This explosion has also brought into sharp focus the impediment presented by the state of transformation technology for many crops [1]. For maize, use of the morphogenic genes *WUS2* and *BBM* has mitigated this bottleneck and has been used in-house for several years for all aspects of our genome modification programs. These include particle-gun-mediated creation of mini-chromosomes [77], CRISPR/CAS9-mediated mutagenesis or editing [78,79], and, of course, random *Agrobacterium*-mediated transformation [17,57]. From our experience with other cereal crops [17] and the progress by Mookkan and colleagues in recalcitrant public lines [56], we feel this technology should make all aspects of genome modification accessible to all cereals, with future enhancements continuing to simplify and improve this approach. Similarly, based on observed morphogenic responses in eudicots and gymnosperms, broadening these methods to include more plant species will hopefully continue to erode the barriers that make so many crops inaccessible for genome editing.

In the foreseeable future, however, finding a single solution that works across all crops is unlikely. Different species and even different varieties within the same species will require new combinations of morphogenic triggers (new combinations of genes or varied expression patterns) to produce either somatic embryos or new apical meristems for rapid production of genetically modified plants. Basic research over the past three decades has provided us with a detailed understanding of the genes that control morphogenesis, and the signaling networks that are so critical to meristem and embryo development, with new insights constantly being discovered. These insights will continue to provide the inspiration for testing morphogenic genes (or combinations of genes) and, along with new strategies to control or limit expression, will result in continued improvements that expand the range of plant species amenable to transformation. Hopefully, this will make plant transformation much more efficient, routine, and accessible for all crops of interest, and will alleviate this key bottleneck to crop improvement, enabling CRISPR/CAS-mediated genome modification in many important crops.

## Figures and Tables

**Table 1:** Strategies to improve transformation using morphogenic genes.

Examples					
Strategy	CDS	Promoter for Transgene	Transformed Species	Variety *	Ref.
(A) Enhance pre-existing somatic embryogenic culture response	*AtSERK1*	35S	*Arabidopsis thaliana*	Ws	[18]
	*CcSERK1 (or RNAi)*	35S and Inducible	*Coffea canephora*	cv. Robusta	[19]
	*AtAGL15*	35S	*A. thaliana*	Ws	[20]
	*GmAGL15*	35S	*Glycine max*	“Jack”	[21]
	*GhAGL15*	35S	*Gossypium hirsutum*	cv. CRI24	[22]
	*AtWUS*	Inducible	*C. canephora*	cv. Robusta	[23]
	*BnSTM*, *BoSTM*	35S	*A. thaliana*	Col	[24]
	*BnSTM*, *BoSTM*	35S	*Brassica napus*	cv. Topas	[24]
	*BrSTM*	35S	*A. thaliana*	Col	[24]
	*BrSTM*	35S	*B. napus*	cv. Topas	[24]
	*AtWUS*	35S	*G. hirsutum*	var. Coker 310	[25]
(B) Ectopic formation of somatic embryos or meristems	*BnBBM*	35S	*A. thaliana*	Col and C24	[26]
	*AtBBM~GR*	Inducible	*Nicotiana tabacum*	Wisconsin 38	[27]
	*AtBBM~GR*	Inducible	*N. tabacum*	Petit Havana SR1	[27]
	*GmBBM*	35S	*A. thaliana*	not specified	[28]
	*TcBBM*	35S	*Theobroma cacao*	Scavina-6 (SCA6)	[29]
	*EgBBM*	35S	*A. thaliana*	Col	[30]
	*AtEMK*	35S	*A. thaliana*	Col-0	[31]
	*AtRKD4*	Inducible	*P.sp* (Orchid)	“Sogo Vivian”	[32]
	*AtLEC1*	35S	*A. thaliana*	Ws-0	[33]
	*CsL1L*	35S	*Clonorchis sinensis*	cv. “Olinda”	[34]
	*PaHAP3A*	Inducible	*Picea abies*	cell lines 88 and 61	[35]
	*AtFUS3*	AtML1	*A. thaliana*	Col	[36]
	*AtLEC2*	35S	*A. thaliana*	Ws-0	[37]
	*AtWUS*	Inducible	*A. thaliana*	Col, Ws, L*er*	[38]
	*AtWUS*, *AtSTM*	Both inducible	*A. thaliana*	L*er*	[39]
	*AtWUS*	Activated	*A. thaliana*	L*er*	[40]
	*AtWUS*	Inducible	*N. tabacum*	cv. Samsun	[41]
	*AtWOX5*	Inducible	*N. tabacum*	cv. Samsun	[42]
	*ZmKN1*	35S	*N. tabacum*	cv. Xanthi	[43]
	*NtKN1*	35S	*N. tabacum*	cv. Samsun	[44]
	*AtCUC1*, *AtCUC2*	35S	*A. thaliana*	L*er*	[45]
	*AtLEC2*	Inducible	*A. thaliana*	Col-0	[46]
	*AtESR1*	Inducible	*A. thaliana*	Ws	[47]
	*AtESR2*	Inducible	*A. thaliana*	L*er* and Ws	[48]
	*AtMPΔ*	MP Promoter	*A. thaliana*	Col-0	[49]
(C) Restrict morphogenic response to enable recovery of normal plants	*BnBBM*	Inducible	*Capsicum annuum*	Three hybrids ^b^	[50]
	*AtBBM*	Inducible	*A. thaliana*	RDL and L*er*	[51]
	*AtPGA37*	Inducible	*A. thaliana*	Col-0, Ws, L*er*	[52]
	*AtLEC2*	Inducible	*T. cacao*	var. SCA6	[53]
	*AtWOX2 WOX8 WOX9*	Inducible	*N. tabacum*	cv. Samsun	[54]
	*BcBBM* ^a^	35S	*Populus tomentosa*	not specified	[55]
	*ZmBBM*/*ZmWUS2*^a^	Ubi + NOS	*Zea mays*	4 Pioneer Inbreds ^c^	[17]
	*ZmBBM*/*ZmWUS2*^a^	Ubi + NOS	*Oryza sativa*	(indica) cv. IRV95	[17]
	*ZmBBM*/*ZmWUS*2 ^a^	Ubi + NOS	*Sorghum bicolor*	var. Tx430	[17]
	*ZmBBM*/*ZmWUS*2 ^a^	Ubi + NOS	*Salvia officianalis*	var. CP01-1372	[17]
	*ZmBBM*/*ZmWUS*2 ^a^	Ubi + NOS	*Z. mays*	public inbred B73	[56]
	*ZmBBM*/*ZmWUS*2 ^a^	Ubi + NOS	*S. bicolor*	var. P898012	[56]
	*ZmBBM*/*ZmWUS*2	PLTP + AXIG1	*Z. mays*	maize inbreds ^d^	[57]

* “Variety” = ecotype (RDL, Wassilewskija = Ws, Columbia = CoL, Landsberg *erecta* = L*er*), variety (var.), cultivar (cv.), inbred, or hybrid name; ^a^ In column labelled “CDS”, these included recombinase-mediated excision for removal of morphogenic gene(s); ^b^ Orchid hybrids Fiesta, Spirit, and Ferrari; ^c^ Pioneer inbreds PHN46, PH581, PHP38, and PHH56; ^d^ Public maize inbreds B73, Mo17, and the FFMM line A (Fast Flowering Mini-Maize, line A). Pioneer inbreds PHR03, PH184C, PHH5G, PH1V5T, and PH1V69.
